# Influence of Waste Glass Particle Size on the Physico-Mechanical Properties and Porosity of Foamed Geopolymer Composites Based on Coal Fly Ash

**DOI:** 10.3390/ma16052044

**Published:** 2023-03-01

**Authors:** Celina Ziejewska, Agnieszka Grela, Marek Hebda

**Affiliations:** 1 Faculty of Materials Engineering and Physics, Cracow University of Technology, Warszawska 24, 31-155 Cracow, Poland; 2 Faculty of Environmental and Power Engineering, Cracow University of Technology, Warszawska 24, 31-155 Cracow, Poland

**Keywords:** geopolymer, waste glass, particle size, compressive strength, leachability

## Abstract

In order to protect the environment and counteract climate change, it is necessary to take any actions that enable a reduction in CO_2_ emissions. One of the key areas is research focused on developing alternative sustainable materials for construction to reduce the global demand for cement. This work presents the properties of foamed geopolymers with the addition of waste glass as well as determined the optimal size and amount of waste glass for improving the mechanical and physical features of the produced composites. Several geopolymer mixtures were fabricated by replacing coal fly ash with 0%, 10%, 20%, and 30% of waste glass by weight. Moreover, the effect of using different particle size ranges of the addition (0.1–1200 µm; 200–1200 µm; 100–250 µm; 63–120 µm; 40–63 µm; 0.1–40 µm) in the geopolymer matrix was examined. Based on the results, it was found that the application of 20–30% of waste glass with a particle size range of 0.1–1200 µm and a mean diameter of 550 µm resulted in approximately 80% higher compressive strength in comparison to unmodified material. Moreover, the samples produced using the smallest fraction (0.1–40 µm) of waste glass in the amount of 30% reached the highest specific surface area (43.711 m^2^/g), maximum porosity (69%), and density of 0.6 g/cm^3^.

## 1. Introduction

Global economic development and perpetual growth in the world population are both connected with a continuing increase in the demand for food, water, and materials, especially for the construction industry. The worldwide annual production of cement reached 4.4 billion tons and 1.39 billion tons in 2021 and 1995, respectively [1,2]. Thus, the production of this material has increased more than threefold in a matter of 26 years and it has been estimated that 3 tons of concrete are manufactured each year per every person around the world [3]. Moreover, global warming and climate change are considered the most significant environmental issues of this millennium. Cement production, due to the decomposition of calcium carbonate (CaCO_3_) into lime (CaO), is one of the main sources of carbon dioxide (CO_2_) emissions, and therefore has a significant impact on climate change [4]. It is estimated that it generates approximately 7% of entire worldwide CO_2_ anthropogenic emissions [5]. Therefore, there is an immediate need to develop new sustainable materials that will cause an effective reduction in the amount of the most common greenhouse gas, CO_2_ emitted into the atmosphere [6]. Furthermore, this assumption is consistent with requirements imposed by the European Commission, which decided to reduce greenhouse gas emissions by at least 40% by 2030 compared to the level of 1990 [7].

Another important issue and serious problem nowadays is the storage of an enormous amount of post-process waste derived from extractive, metallurgical, and power industries. Their landfill may result in ecological problems due to the impact of pollutants on soils, surface water, and groundwater. In addition, it requires financial outlays, as well as poses a risk of self-ignition. Fly ash ranks among the most substantial waste from the furnaces in coal combustion plants. Polish industry generates about 5 million tons of fly ash annually, with only a small part of it recycled [8,9].

Therefore, the prospect of using such waste products after processing as a base material for the production of geopolymers should be emphasized [10]. Geopolymer is an inorganic polymeric material obtained from silica-aluminate materials, such as metakaolin [11], fly ash [12], silica fume [13], clay [14], and red mud [15] by the geopolymerization process [16]. The geopolymer primary structure consists of [SiO_4_]^4−^ and [AlO_4_]^5−^ anions linked by an oxygen atom [17]. Geopolymers are currently becoming increasingly popular in the scientific community as well as the construction industry due to their properties, such as high fire resistance (they show stability up to 1000–1200 °C) [18], great mechanical properties, including good compressive (even more than 100 MPa) and flexural strength (up to 25 MPa) [19], frost resistance (even at the level of F300) [20], excellent dimensional stability [21], and acid resistance [22]. However, apart from the technical issues offered by geopolymers, their positive impact on the environment should also be considered. Geopolymerization is a low-cost technology, which enables the use of waste materials, reduces energy consumption, as well as decreasing the total carbon dioxide footprint because high temperatures are not required in the geopolymer manufacturing process and therefore CO_2_ emissions are reduced by around 70% compared to in the process to manufacture Ordinary Portland Cement (OPC) [23,24,25]. Due to their properties, geopolymers are becoming more and more widely used in various industries, in applications such as materials capable of immobilization toxic substances [26], construction materials [27], structural materials [28], and protective coatings [29].

Glass is a widely used material all over the world [30] because of its properties, such as transparency [31] and chemical durability [32]. According to the literature data, the total global production of glass reached approximately 89.4 Mt in 2007 [33]. However, many end-of-life glass products or glass waste are landfilled [34,35]. The global recycling rate of waste glass reached only 21% in 2018 [36], whereas in China it was about 50% in 2021 [37] and Australia achieved 59% in 2020–2021 [38]. The rest of the material has been continuously accumulating in landfills for years because it is a non-flammable and non-biodegradable material. Waste glass may be reused in the glass industry, however, an insufficient quantity of reused glass results from, among other things, requirements for the quality of the raw material, which is necessary to obtain high-quality products. Moreover, multicolored waste glass might not meet requirements in regards to its properties after the deinking process, making it difficult to recycle it into new glass products. However, the use of recycled glass would contribute to reducing landfill volumes, managing waste, reducing CO_2_ emissions, protecting the energy required to melt glass, and saving natural resources [39,40,41]. It was found that the addition of 10 % of glass cullet in the furnace during glass manufacturing decreases energy consumption by 3%, as well as CO_2_ emissions by around 5%.

As waste glass contains large amounts of silica and alumina, it may be an alternative source of building materials. Studies indicate the possibility of using waste glass for geopolymer production [33,39]. The addition of waste glass powder can positively affect the mechanical properties of geopolymers [28,33,42,43], or their fire resistance [8]. Additionally, glass cullet can be used to produce geopolymer foams [41,42,44], as well as nonporous materials [45,46].

Waste glass is commonly used as an additive in concrete. The most beneficial effect is achieved through the application of waste glass with a particle size of 75 µm [47]. In general, there is a tendency to use waste glass characterized by small particle sizes, as a pozzolan and fine aggregate in concrete manufacturing [48]. Shi et al. [49] stated that the pozzolanic activity of waste glass is higher the finer particle size is. However, there is still a research gap relating to the application of waste glass in geopolymer foam.

In general, one of the most popular methods for producing geopolymer foam with glass is the application of the sintering process. Badanoiu et al. [50] investigated geopolymer foam based on red mud and cullet obtained using thermal treatment at 600–800 °C for 1 h. Similarly, other authors [41] used temperatures ranging from 600 °C to 750 °C for 1 h during the manufacture of geopolymers with waste glass particle sizes of 23 and 72 µm, whereas Tramontin Souza et al. [51] used temperature treatment at 900 °C for 30 min. Moreover, Siddika et al. [52] applied a sintering process for geopolymer with a particle size of D50 = 25 µm waste glass for 10–60 min at 800 °C. However, high-temperature treatment is associated with high energy consumption and carbon dioxide emissions. Therefore, there is a possibility to obtain geopolymer foam with waste glass without using the sintering process. Zhang et al. [53] explored geopolymer foams with three particle sizes of waste glass, D50 = 49.2 µm, 159.1 µm, and 302.1 µm, cured at 20–100 °C, and found that finer particles influenced the higher level of geopolymerization and improved compressive strength. Ruan et al. [54] proved that aluminium powder is a suitable foaming agent for geopolymers obtained at low temperatures (80 °C). However, to date, no study has focused on the impact of the percentage content and particle size of five different fractions of waste glass in the range between 0–1200 µm on the properties of produced foamed geopolymer composites, obtained without the application of a high-temperature treatment, and the presented work purposes complement the existing lack of information. Furthermore, researchers in previous studies [33,44,55] had used only glass waste after cleaning, without contamination. However, these proceedings required water and energy consumption, as well as having a negative impact on the environment due to the wastewater generated. Wang et al. [44,56] studied the influence of contaminated waste glass fines on the concrete behavior at 10 wt% substitutions of sand. It was found that the application of such a quantity of unwashed waste glass does not cause a higher environmental risk than the use of traditional concrete. Therefore, in the present work, unwashed waste glass with different particle diameters was used in the range of: 0.1–1200 µm; 200–1200 µm; 100–250 µm; 63–120 µm; 40–63 µm; 0.1–40 µm, with a weight fraction of from 0 to 30%, was used as an additive to geopolymers to evaluate the effect of the applied amount on the properties of produced samples. Density, porosity, specific surface area, mineralogical composition, morphology, leachability, water absorption, and mechanical properties, such as flexural strength and compressive strength were investigated. Moreover, the effect of partially replacing river sand and coal fly ash with waste glass was described.

## 2. Materials and Methods

### 2.1. Materials

Coal fly ash (with the chemical composition presented in Table 1) was supplied by the Skawina Coal Power Plant (Skawina, Poland) and it was labeled as Class F fly ash in accordance with the ASTMC618 standard [57]. The loss of ignition (LOI) of coal fly ash was 3.284 and this parameter is usually used to evaluate the residual carbon content [58]. The quartz sand was supplied by an indigenous company (Świętochłowice, Poland). Sodium silicate (Na_2_SiO_3_) R-145 was purchased from Chemi Kam sp. Z o.o. (Będzin, Poland). The waste glass (WG) that was applied in this study (with the chemical composition shown in Table 1) was sourced from a local supplier Grabowski Import-Export (Sędziszowa, Poland). The waste glass was derived from unserviceable brown bottles. The company crushed and ground the glass waste to obtain particles size smaller than 12 mm. Using a set of sieves and a laboratory shaker, the delivered glass waste was divided into fractions: 0.1–1200 µm; 200–1200 µm; 100–250 µm; 63–120 µm; 40–63 µm; 0.1–40 µm.

### 2.2. Samples Preparation

To provide a homogeneous mixture, all dry components (coal fly ash, waste glass, sand) were mixed for 2 min in a GEOLAB cement mortar mixer (GEOLAB, Warsaw, Poland). Alkali activator solution was then added to the starting materials. A mix of sodium hydroxide solution of 8 M and an aqueous solution of sodium silicate was used as an alkaline activator in a proportion of 2.5:1. The solution was prepared 24 h before use to provide complete mixing of the ingredients and reach a constant temperature. The liquid-to-solid ratio (L/S) was set at the level of 0.4 to get proper workability. The final step before the casting of samples was adding aluminum powder (5-7350 type, Benda-Lutz, Skawina, Poland) as a foaming agent (0.15% by weight). Mixtures were then put into wooden molds of the appropriate shapes and sizes. Geopolymer samples were heated in the drying apparatus (Chemland) for 24 h at 75 °C. After demolding, samples were cured at ambient conditions for 28 days.

Varied weight ratios and different particle sizes of waste glass were added to the geopolymer mixture to evaluate the effect of the used addition on the properties of the samples. The compositions of geopolymer mixtures were fixed based on the previous studies, which indicates that waste glass should be applied in quantities of 10% to 30% [47,59,60]. Therefore, three different weight ratios of waste glass were used in geopolymers: 10%, 20%, and 30%, which correspond to the calculated theoretical SiO_2_/Al_2_O_3_ mole ratios of 5.19, 6.07, and 7.22, respectively. As a reference, a sample without added glass with a theoretical SiO_2_/Al_2_O_3_ molar ratio of 4.48 was used. Moreover, the Na_2_O/SiO_2_ molar ratios were 0.14, 0.15, 0.16, and 0.17 for samples with 0%, 10%, 20%, and 30% of waste glass, respectively. The composition of the designed geopolymer samples is given in Table 2.

### 2.3. Analytical Methods for Raw Materials and Geopolymers Characterization

The mineralogical composition of the raw materials and geopolymers was determined by a PANalytical Aeris diffractometer (Malvern Panalytical, Almelo, The Netherlands) using Cu Kα radiation, scanning range from 10° to 100° 2θ, step size 0.003° (2θ), and measurement time per step of 340 s. High Score Plus software version 4.8 (PANalytical) equipped with the ICDD (International Center for Diffraction Data, PDF4+) database was used to identify the diffraction patterns.

The particle size distribution of the raw materials was measured using a Particle Size Analyser PSA 1190 LD (Anton Paar, Graz, Austria).

The morphology of the raw materials and geopolymers samples were examined using a Keyence VHX-E100 digital microscope (Keyence, Osaka, Japan) as well as a scanning electron microscope JEOL JSM-6390LV (JEOL, Tokyo, Japan).

The specific surface area of the raw materials and geopolymers was determined by the Brunauer–Emmett–Teller (BET) gas adsorption method. Before registering adsorption-desorption isotherms, the specimens were degassed at 300 °C for 24 h to remove expendable vapors and gases adsorbed on the sample surface. Nitrogen adsorption-desorption isotherms of the investigated materials were recorded using Autosorb-iQ/MP Quantachrome Instruments gas sorption analyzers (Anton Paar company, Graz, Austria).

The density of the produced geopolymers was calculated by the geometric method as the ratio of the mass to the volume of the samples. The masses of the specimens were measured using a Radwag XA 60/220/Y balance (RADWAG, Radom, Poland).

The samples intended for use in testing the mechanical properties had dimensions of 50 mm × 50 mm × 50 mm, and 200 mm × 50 mm × 50 mm for the compressive and flexural strength tests, respectively. Compressive strength tests were carried out according to the PN-EN 12390-3:2019 standard using a MATEST 3000 kN machine (MATEST S.p.A., Arcore, Italy). Flexural strength tests were performed in accordance with the PN-EN 12390-5:2019 standard on a concrete press (MATEST S.p.A., Arcore, Italy).

The leaching assessment was conducted according to the PN-EN 12457-4:2006 standard. The pH of the water extract was analyzed by the potentiometry method, at a temperature of 21.9–23.2 °C. The concentrations of zinc, cadmium, copper, lead, nickel, barium, chromium, arsenic, selenium, molybdenum, and antimony were determined by Inductively Coupled Plasma Optical Emission Spectrometry (ICP-OES). The dissolved organic carbon (DOC) content of samples was characterized by Fourier Transform Infrared Spectroscopy (FTIR). Determination of the total dissolved solids (TDS) was made by the gravimetric method. The mercury concentration was defined by Cold Vapor Atomic Absorption (CVAA) spectroscopy. The SO_4_^2−^ and Cl^−^ ion content was measured using the ion chromatography method.

The water absorption tests were conducted in accordance with PN-88/B-06250 “Ordinary concrete” on 5 cm geopolymer cubes. The following relationship was used:

(1) $$n_{w}=\frac{G_{2}-G_{1}}{G_{1}}\cdot 100\;[\%]$$

where $n_{w}$ is water absorption; $G_{1}$ is the average mass of dry samples; and $G_{2}$ is the average mass of the samples saturated with water.

ImageJ software version 1.53t was used to calculate the porosity, average cell size, and cell density of materials, using 2D photographs of geopolymer structures.

The average cell density of samples was determined based on the following equation:

(2) $$N={\left[\frac{\left(nM^{2}\right)}{A}\right]}^{\frac{3}{2}}\;[\mathrm{cells}/{\mathrm{cm}}^{3}]$$

where *N* is the cell density; *n* is the number of cells in the SEM image; *A* is the area of the analyzed image; and M is the magnification [61].

Regardless of the research method used and the type of materials analyzed, the measurements were performed with at least three repetitions.

## 3. Results and Discussion

### 3.1. Properties of Raw Materials

**Figure 1:**
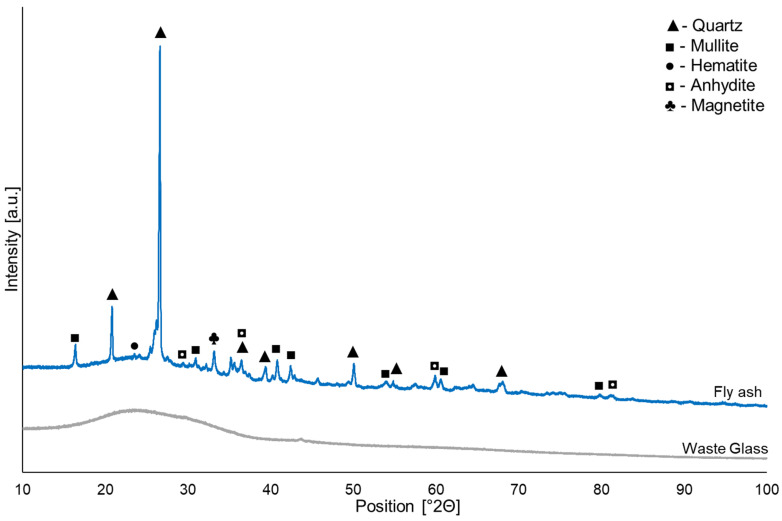
XRD patterns of waste glass and coal fly ash.

X-ray diffraction (XRD) patterns of the coal fly ash and glass waste used in the experiment as raw materials are shown in Figure 1. The qualitative X-ray diffraction analysis of coal fly ash enabled the identification of the following crystalline phases: quartz (SiO_2_ card no.: 01-089-8936), mullite (Al_6_Si_2_O_13_, card no.: 00-015-0776), hematite (Fe_2_O_3_, card no.: 01-079-0007), anhydrite (CaSO_4_, card no.: 01-085-6123), and magnetite (Fe_3_O_4_, card no.: 01-080-6407). However, the coal fly ash also contained a broad hump in the range from 15° to 30° 2θ, suggesting the existence of amorphous components, which are primarily in charge of the reactiveness of the raw materials [62]. Quantitative X-ray analysis of the coal fly ash enabled a determination of the content of individual phases, whose shares were respectively: 50.3% SiO_2_, 45.0% Al_6_Si_2_O_13_, 2.1% Fe_2_O_3_, 2.4% CaSO_4_, and 0.2% Fe_3_O_4_. The results of the quantitative analysis can only be considered as approximate values due to the existence of the amorphous phase, the high intensity of background noise, and overlapping reflections. The XRD pattern of the waste glass indicates that this is a completely amorphous material, different from the coal fly ash.

**Figure 2:**
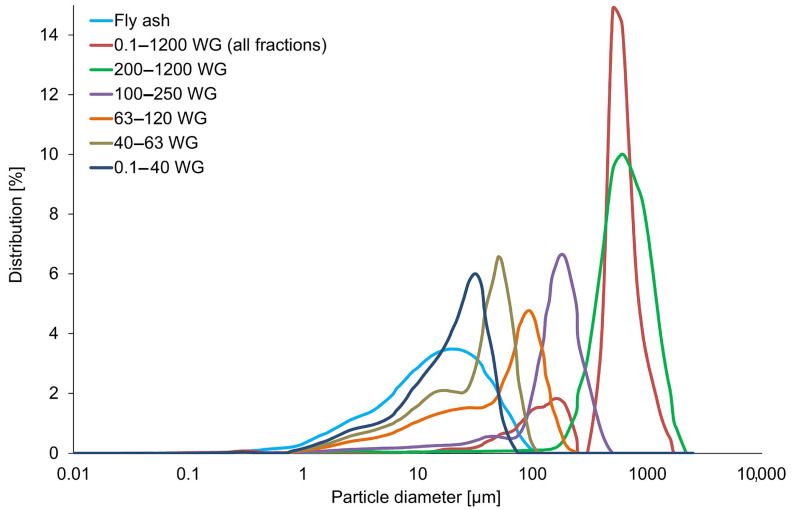
The particle size distribution of coal fly ash and glass waste before and after the division into different fractions.

Figure 2 presents the particle size distribution of the coal fly ash and waste glass before and after separation into different fractions. Coal fly ash has the smallest average particle size among all used starting materials. Moreover, Table S1 in Supplementary Materials demonstrates the averaged results of the size distribution of coal fly ash and waste glass used in this study.

The coal fly ash was characterized by comparable median and mean values (12.3 ± 1.3 µm and 17.3 ± 2.5 µm, respectively) and a small span range, which indicates an approximately normal particle size distribution [57]. Conversely, the as-delivered waste glass is distinguished by higher variations in this regard, with a mean particle size of 550.1 ± 18.9 µm. As can be seen in Figure 2, the particle size distribution curve of the unsorted waste glass consists of two peaks, representing particles about 170 µm and 600 µm in size. Moreover, in the case of the waste glass after division into fractions, the mean particle size was 584.9 ± 4.4 µm, 155.2 ± 0.5 µm, 55.4 ± 1.2 µm, 33.3 ± 0.1 µm, and 19.8 ± 0.3 µm, which was adequate for 0.1–1200 WG, 100–250 WG, 63–120 WG, 40–63 WG, and 0.1–40 WG. The obtained results were in line with expectations.

**Figure 3:**
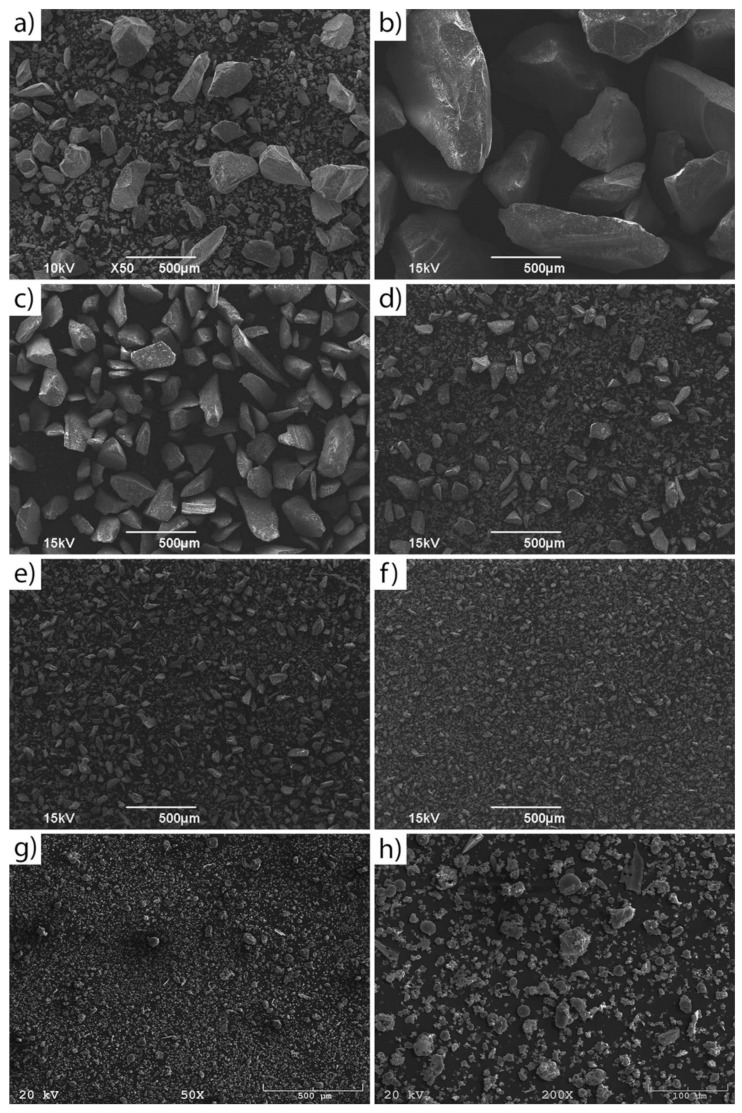
SEM images of waste glass with different particle sizes: (**a**) 0.1–1200 WG (all fractions), (**b**) 200–1200 WG, (**c**) 100–250 WG, (**d**) 63–120 WG, (**e**) 40–63 WG, (**f**) 0.1–40 WG and (**g**,**h**) coal fly ash.

Figure 3a–g illustrates the morphology of the raw materials. The as-delivered waste glass consisted of particles of varying shapes and sizes (Figure 3a). Furthermore, the obtained images also confirmed the effectiveness of sieving concerning the obtained particle size and distribution of waste glass. The coal fly ash consisted of many porous particles of various sizes and shapes, such as spherical, irregular, and angular, but consisted predominantly of spherical particles. Such a morphology of coal fly ash has a beneficial effect on the geopolymerization process, as it enhances its reactivity [63].

**Figure 4:**
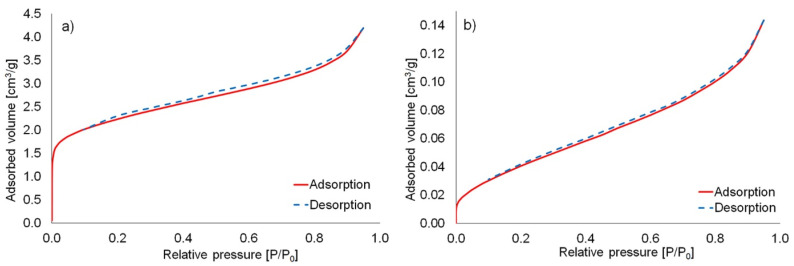
Nitrogen adsorption-desorption isotherms of (**a**) coal fly ash; (**b**) unsorted waste glass.

The nitrogen adsorption-desorption isotherms of coal fly ash and the as-delivered waste glass (all fractions WG) are presented in Figure 4, whereas the isotherms of waste glass after separation into different fractions are shown in Figure S1 in Supplementary Materials. The specific surface area was determined by using the multi-BET method and was found to be 7.804 m^2^/g and 0.152 m^2^/g for coal fly ash and unsorted brown waste glass, respectively. Moreover, the specific surface area of waste glass after separation into five different fractions reached the following values: 0.048 m^2^/g for 200–1200 WG, 0.114 m^2^/g for 100–250 WG, 0.375 m^2^/g for 63–120 WG, 0.594 m^2^/g for 40–63 WG, and 0.693 m^2^/g for 0.1–40 WG. Based on the IUPAC (International Union of Pure and Applied Chemistry) classification, the N_2_ isotherms of coal fly ash and waste glass correspond to type II isotherms with an H3-type hysteresis loop. Type II nitrogen adsorption-desorption isotherm indicates that the investigated material was non-porous or microporous, as well as having a comparatively low surface area [64,65].

### 3.2. Properties of Produced Geopolymers

**Figure 5:**
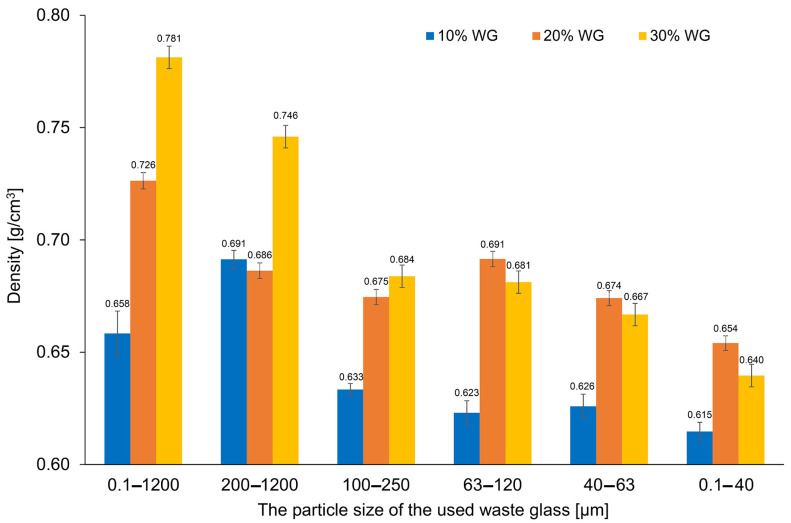
The density of geopolymers depending on the percentage and size of the fraction of the waste glass addition.

Figure 5 shows the density results of the produced geopolymer samples including waste glass determined using the geometrical method after 28 days of seasoning. The reference sample, not containing added waste glass, demonstrated a density of 0.69 ± 0.04 g/cm^3^. On the basis of the obtained results, a visible effect of increasing density with higher content of waste glass was observed. A similar tendency was noticed by other authors [66,67,68,69]. Siddika et al. [47] observed that a higher content of waste glass increased the density and reduced the porosity of cement concrete. In contrast, regarding the effect of the particle size of the waste glass on the density of the materials produced, it was generally found that the use of a smaller additive size resulted in a decrease in geopolymer density. This effect was independent of the proportion of the introduced addition of glass waste to the matrix.

As can be seen, the density of geopolymers with added waste glass with a particle size up to 120 µm (0.1–40 WG, 40–63 WG, 63–120 WG) was the highest for samples containing 20% of waste. Similar findings were obtained by Ahmad et al. [70] who investigated the properties of concrete with the addition of waste glass with a particle size of up to 75 µm. It was concluded that waste glass undergoes a pozzolanic reaction, creating additional C-S-H gel resulting in an increase in the viscosity and density of the blends. At the same time, the higher content of waste glass hinders the compaction process, causing the formation of a larger number of pores and thereby decreasing material density.

The specific surface area (calculated using the BET equation) of the produced geopolymers was significantly higher than that of the raw materials and in the case of the reference sample, without the addition of waste glass, it reached a value of 22.772 m^2^/g. The addition of unsorted waste glass caused an increase in the surface area by up to 65% in the case of the A20 sample. Moreover, it was found that the decrease in the size of the waste glass particles introduced into the matrix resulted in an increase in the specific surface area of the produced composite. Janowska-Renkas et al. [66] confirmed that waste glass with lower particle sizes is characterized by higher surface area. The decrease in particle sizes of waste glass, and therefore the increase in their specific surface area cause increased pozzolanic reactivity [47]. Coal fly ash has a higher surface area than waste glass; therefore, increasing the amount of used waste cullet and consequently reducing the applied coal fly ash in specimens with unsorted waste glass should increase their surface area, which would be entailed by the summation of their properties. However the specific surface area of the A20 and A30 samples amounts to 37.556 m^2^/g and 36.435 m^2^/g, respectively, and, therefore, this trend is not clearly visible here due to the wide and somewhat random range of particle sizes of waste glass or the structure of the samples. All the designated values of BET are shown in Table S2 in Supplementary Materials.

**Figure 6:**
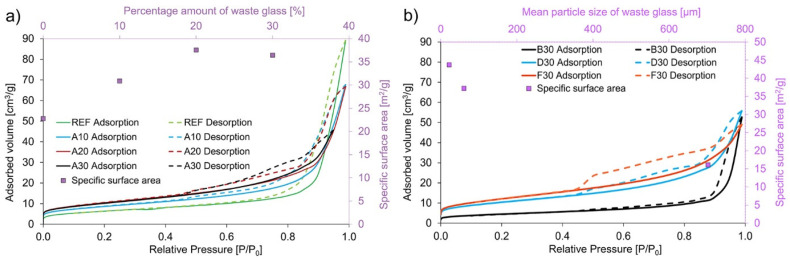
The nitrogen adsorption-desorption isotherms of geopolymers with the addition of waste glass with (**a**) various amounts by weight (0–30%); (**b**) different diameters of particle sizes.

All the obtained adsorption-desorption isotherms (Figure 6) of produced geopolymers based on coal fly ash with the addition of waste glass can be qualified as IV type with an H3 hysteresis loop in compliance with IUPAC categorization [71]. The obtained IV type of adsorption-desorption isotherms is a typical result of the investigation of mesoporous samples with 2–50 nm diameter [72]. The presence of hysteresis is a result of condensation within the capillaries of slit-shaped mesoporous structures [73,74].

**Figure 7:**
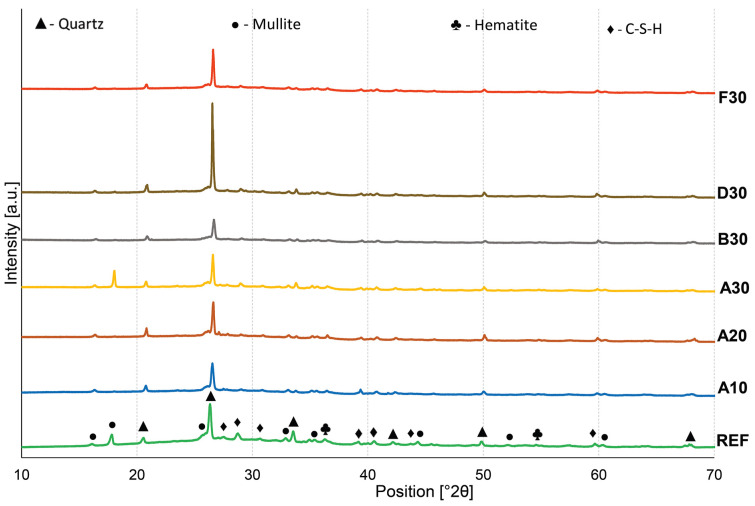
The XRD patterns of geopolymers with varying waste glass content.

The result of various amounts of waste glass addition on the mineralogical composition of the geopolymers is presented in Figure 7. After the geopolymerization process, the obtained samples contained crystallized phases, such as quartz (SiO_2_, card no.: 01-075-8320) as the primary phase occurring in geopolymers because of the substantial silica amount [75], as well as mullite (Al_6_Si_2_O_13_, card no.: 01-082-1237), and hematite (Fe_2_O_3_, card no.: 01-079-0007), which indicate the presence of unreacted elements from the raw materials [76,77]. However, the diffuse broad hump between 20–40° 2θ in all specimens proves the presence of an amorphous component in the form of C-S-H gel (as a Rosenhahnite, card no.: 00-029-0378). This is also confirmation that the geopolymerization process took place [78,79]. C-S-H gel developed due to the adequate content of calcium oxide in the starting materials [80]. On the basis of the obtained results, it can be concluded that the change in the amount of glass waste addition as well as the size of their fractions had a negligible effect on the type of mineralogical composition present in the produced geopolymers. The degree of crystallinity of the geopolymers based on their XRD pattern was also determined, and it reached the following levels: 0.324 for REF, 0.322 for A10, 0.308 for A20, 0.248 for A30, 0.230 for B30, 0.248 for D30, and 0.270 for F30.

**Figure 8:**
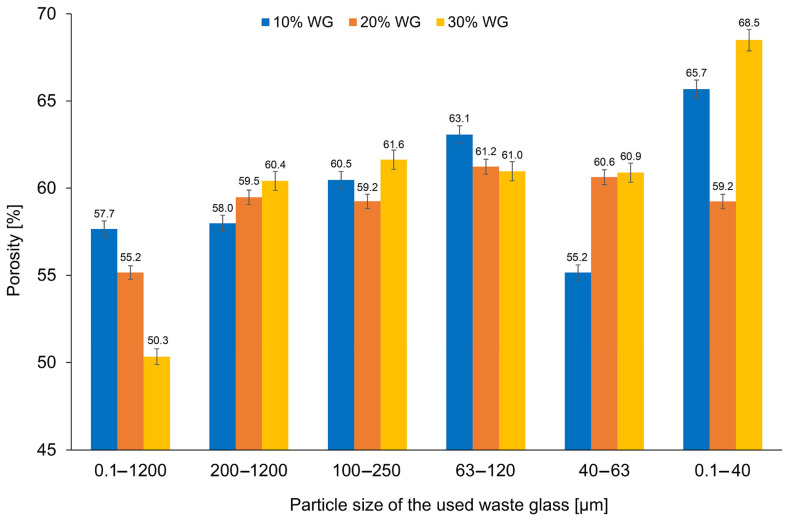
The porosity of geopolymers depending on the percentage and size of the fraction of the waste glass addition.

It is well known that porosity depends on various factors, such as the type and fineness of the foaming agent [81], stabilizer [82], alkali content [83], curing temperature [84], and the type of raw materials [85]. The influence of waste glass on the obtained porosity of geopolymers is shown in Figure 8. An example of the determination of porosity is presented in Figure S2 in Supplementary Materials. In the presented study, the porosity of the geopolymers with waste glass ranged from 50.3% to 68.5%, whereas for reference specimens it was 50.5%. The application of unsorted waste glass in the amount of 30% resulted in a reduction in porosity of 9.5% in comparison to the reference material; while reducing the amount of additive introduced to 20% or 10% caused the opposite effect.

**Figure 9:**
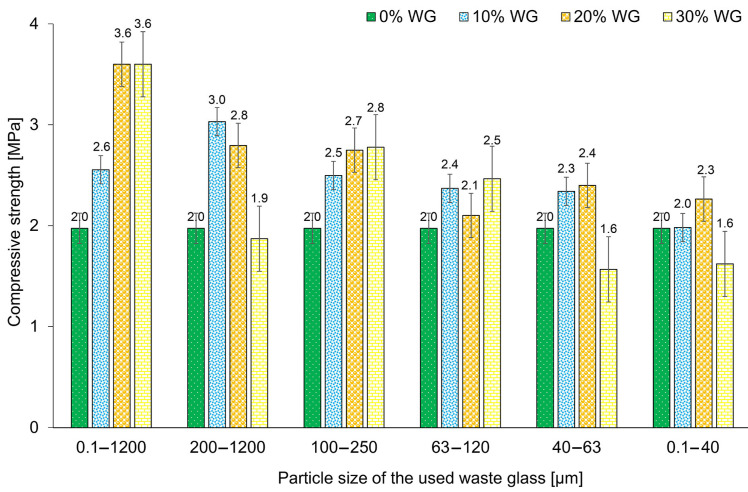
The compressive strength of geopolymers depending on the percentage and size of the fraction of the waste glass addition, after 28 days of curing.

The effect of waste glass on the compressive strength of the geopolymers is shown in Figure 9. On the basis of the results, it was noticed that the highest results were obtained for foams containing unsorted waste glass. The addition of 20% as well as 30% of unsorted glass waste to the geopolymer increased the compressive strength of samples by 80% compared to the reference specimens, tested after 28 days of curing. In general, the higher compressive strength of geopolymers with the addition of waste glass may result from a more efficient pozzolanic reaction due to higher access to dissolved aluminum and silica [30]. Moreover, the application of this type of waste glass is also an environmentally and economically friendly solution due to the elimination of the necessity to use additional processing such as grinding or crumbling. At the same time, it should be noted that the use of the addition of waste glass in the same proportion but with a different particle size may result in obtaining different effects than for unsorted glass. For example, the use of waste glass with a particle size of up to 63 µm, initially up to 20% of the additive, increases the compressive strength, however, the samples containing 30% of the additive showed a decrease in properties even below the values obtained for the reference materials.

The obtained results for compressive strength are consistent with the results of porosity presented earlier. Generally, it can be stated that increasing the porosity of the geopolymers decreased the compressive strength. A similar observation was made by Deng et al. [86], who noticed that the increase in strength was related to the filling of the existing porosity in the material by glass additive.

**Figure 10:**
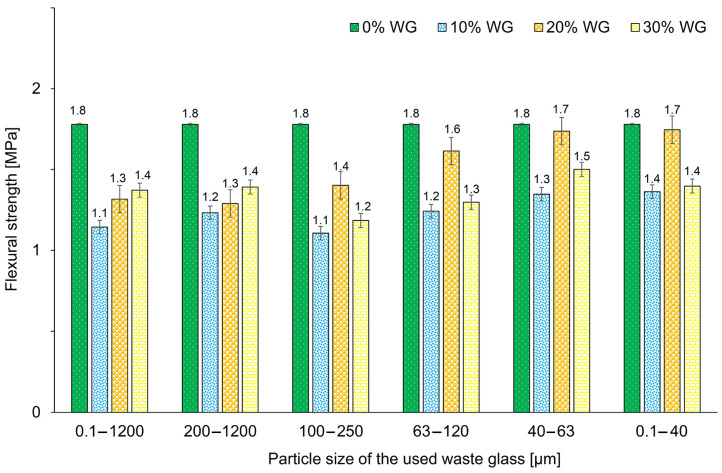
The flexural strength of geopolymers depending on the percentage and size of the fraction of the waste glass addition, after 28 days of curing.

The influence of waste glass content and particle size on the flexural strengths of geopolymers is presented in Figure 10. The incorporation of waste glass reduced the flexural strength of all samples, regardless of the particle size and amount of waste glass used for geopolymer synthesis. A similar effect was noticed by Ali et al. [87] and Toniolo et al. [88] in their studies. However, the effect of waste glass particle size is clearly visible here. The flexural strengths of samples tended to increase along with the application of smaller particle sizes of the introduced additive.

Siddika et al. [47] indicated that the particle size of waste glass should be in the range of 38–75 µm to obtain the optimal value of pozzolanicity as well as silica dissolution in concrete. When considering the influence of waste glass with particle sizes ranging from 100–250 µm, 63–120 µm, 40–63 µm, and 0.1–40 µm on the flexural strength, the highest values were reached for 20% content of the cullet. A possible explanation for this phenomenon is the developing value of the Na_2_O/SiO_2_ ratio in the geopolymers. After exceeding the optimal value, the additional Na^+^ ions may have resulted in excessive efflorescence, as well as a strength decrease [89]. The specimens containing bigger particle sizes of waste glass (0.1–1200 WG and 200–1200 WG) present an increasing trend in flexural strength with increasing incorporation of waste from 10% to 30%, but the obtained results are significantly lower than the reference material. Tahwia et al. [60] noticed that the incorporation of waste glass may result in the appearance of empty voids within the material due to their particle angularity and this may result in reduced mechanical properties. Even though the flexural strengths of the specimens are decreased due to the incorporation of waste glass, it should be noticed that samples included 20% of cullet with a particle size of up to 0.1 mm (D20, E20, F20) have results close to the values of the reference sample.

Based on the results obtained it can be concluded that the use of waste glass can significantly influence the mechanical properties of geopolymers by reducing their flexural strength and simultaneously increasing their compressive strength. Moreover, it was noticed that there is no correlation between compressive strength and flexural strength [90]. A similar effect, which is the opposite of the relations occurring in concrete technology in which compressive strength is associated with flexural strength, was also observed by other researchers [91,92,93]. The different fracture processes in both types of loads could be the reason for this phenomenon. Moreover, in the presented study, foamed samples were investigated. The pore size and their distribution affect the mechanical properties of geopolymer, as well as the material’s fracture resistance. Furthermore, the distribution of the additive has a crucial impact on the fracture properties of geopolymers.

Leaching test results from the geopolymer samples are presented in Table S3 in Supplementary Materials. Introducing a supplement in the form of waste glass particles into geopolymers proved to have an insignificant influence on the content of heavy metals such as Hg, Cd, Ni, Cr, Cu, Zn, and As in the leachates. Only the content of Pb is higher for geopolymers with the addition of the lowest particle size of waste glass than in the case of the reference material. The higher Pb content in the F30 leachates resulted from the dissolution of metals from the glass particle surface, which increases with the reduction in the particle size (Table S2 in Supplementary Materials) [94]. Shi et al. [95] noticed a similar tendency that, with increasing particle size, the content of Pb decreases, and it was concluded that it is related to the type of additives in the geopolymer structure. Bobirică et al. [96] investigated the leaching behavior of geopolymers with the addition of waste glass obtained from worn linear fluorescent lamps. It was found that Pb was leached in a larger amount in the case of geopolymers characterized by finer particle sizes.

The content of all examined hazardous metals in geopolymers was within the range of leaching limit values for inert waste in compliance with European Council Decision 2003/33/EC [57,97] except for the level of total dissolved substances, which is beyond the range of values for inert waste. However, it is below the limit of non-hazardous waste. It can be associated with the porosity and density of the produced geopolymers. Within samples with 30% of various sizes of waste glass, the highest concentration of TDS was reached for F30, which has the highest porosity and smallest density.

**Figure 11:**
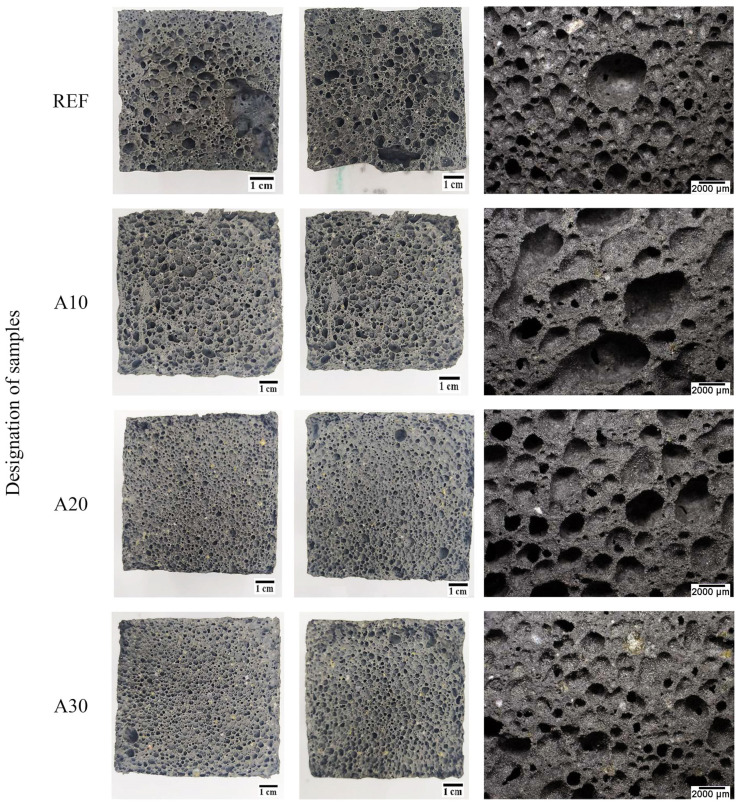
The structure of the geopolymer with waste glass with a particle size of 0.1–1200 µm, depending on the waste glass content.

**Figure 12:**
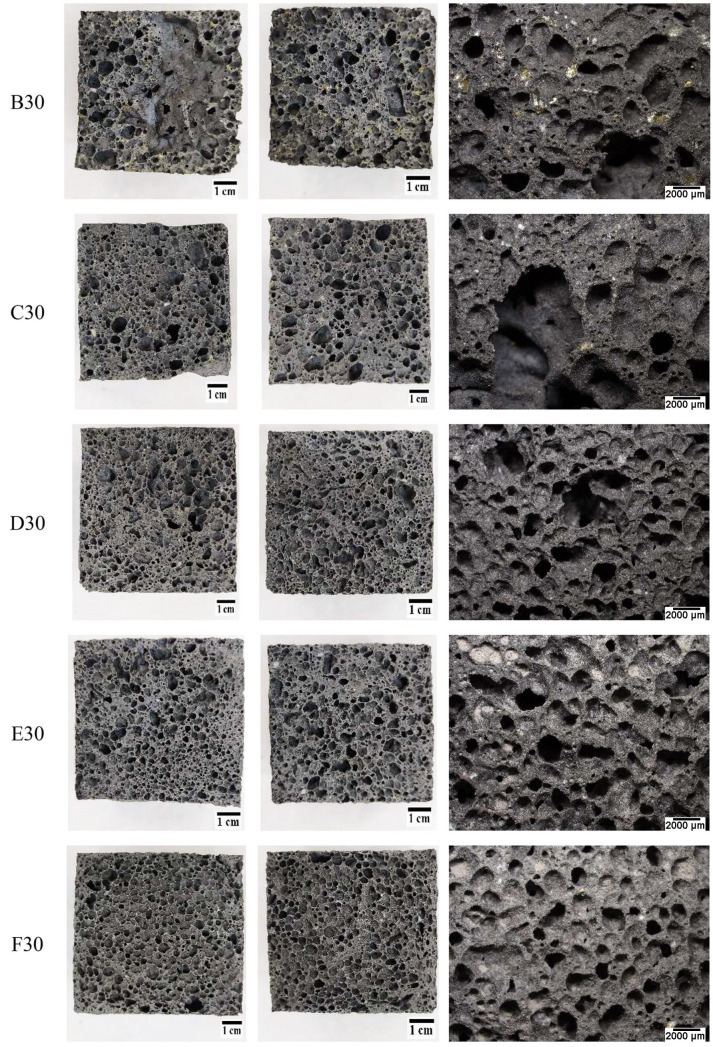
The structure of the geopolymer with the 30% addition of waste glass, depending on the particle size fraction.

The influence of the content of unsorted waste glass (with particle size from 0.1 µm to 1200 µm) on the geopolymer structure depending on the percentage is presented in Figure 11, while the effect of the particle size for the samples containing 30% of the waste glass addition is presented in Figure 12. It was found that, with an increase in the content of the unsorted waste glass, the pores had smaller diameters and were more evenly distributed in the geopolymer structure. Moreover, the addition of waste cullet has a beneficial influence on the homogeneity of the porous structure of the samples. It is well known that porosity has a fundamental influence on a material’s compressive strength [98]. Samples without the addition of waste cullet have large porous macrostructures, which can cause deterioration of mechanical properties. The pictures presented in Figure 12 show that a significant amount of large-sized pores was generated as a result of the application of waste glass with larger diameters. In general, the size of the pore reduces with the reduction in waste glass particle size.

**Figure 13:**
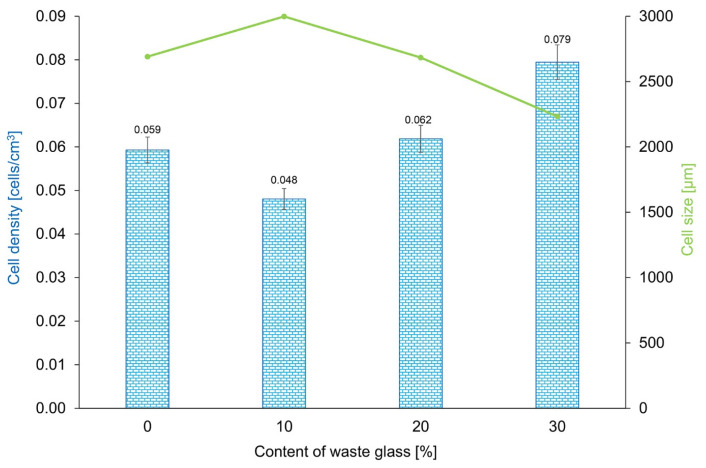
Cell size and cell density of geopolymers with the addition of waste glass with various amounts by weight (0–30%).

The correlation between cell density and cell size is presented in Figure 13. In general, with the increase in the introduced waste glass, the cell size of geopolymers decreased. However, an opposite relationship existed between cell density and waste glass content, and with increasing waste glass addition, cell density also increased. A similar tendency was also noticed by Fei et al., who proved that introducing the additive into a polymer resulted in a larger average cell size and smaller cell density of the investigated material [99]. On the basis of the presented results for geopolymers with different percentage amounts of waste glass (D50 = 483.4 µm), it can be concluded that samples with 30% of WG have the highest density (0.079 cells/cm^3^) and the smallest cell size (2233 µm).

**Figure 14:**
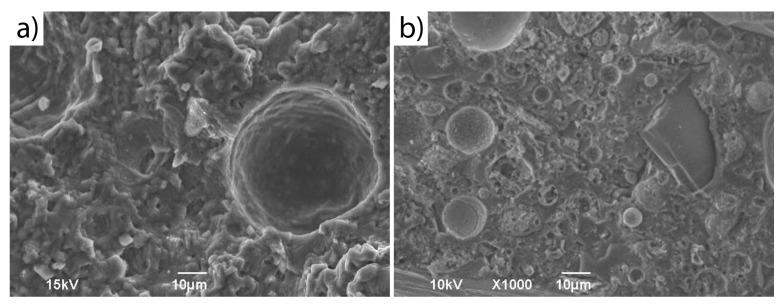
SEM microstructure of geopolymer samples: (**a**) REF; (**b**) A30%.

Representative SEM images of foamed geopolymers with 0% and 30% content of unsorted waste glass (0.1–1200 WG) are shown in Figure 14. It was noticed that the reference geopolymer sample had a homogeneous porous compact microstructure. However, in the geopolymer microstructure with the addition of glass waste, one can observe spherical particles of unreacted coal fly ash and particles of undissolved waste cullet (with irregular shapes, smooth surfaces, and sharp edges) in the geopolymer matrix. The presence of such undissolved glass particles influenced the leachability results described earlier.

**Figure 15:**
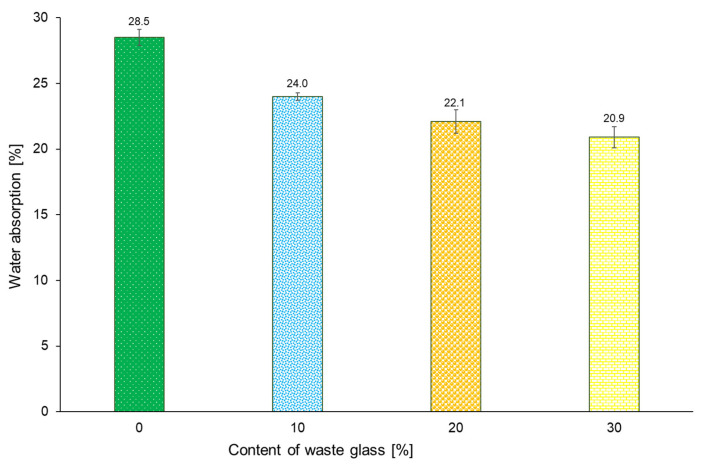
Water absorption of geopolymer samples containing varying amounts of unsorted waste glass.

Figure 15 shows the water absorption results of the geopolymer samples containing varying amounts of waste glass. In general, water absorption depends on the voids content present in the examined materials [100]. On the basis of the obtained results, it was found that the water absorption of the geopolymers tends to decrease with increasing waste glass content. Thus, the addition of waste glass to the geopolymer matrix fills the pores formed, changes the porosity morphology, and influences capillary processes; it also increases the packing of particles in the geopolymerization process [101]. For the geopolymers with 10%, 20%, and 30% of unsorted waste glass, the decrease in water absorption relative to the reference sample was 15.8%, 22.5%, and 26.7%, respectively.

## 4. Conclusions

For the first time, the impact of content and particle size of the addition of waste glass ranged between 0–1200 µm and divided into five fractions on foamed geopolymer composites was characterized. The density, specific surface area, mineralogical composition, compressive strength, flexural strength, leachability, water absorption, and porosity of foamed geopolymers were determined.

Based on the presented results, it was found that the content and particle size of waste glass both have a crucial impact on the porosity of the geopolymers. The formation of smaller, evenly distributed pores in the geopolymer structure can be achieved by increasing the content of waste glass with a particle size of 0.1 to 1200 µm or reducing the size of the added waste glass particles. The highest porosity (68.5%) was achieved by samples with 30% of waste glass with the smallest particle sizes (0.1–40 µm). Moreover, an increase in the weight share of waste glass, as well as a decrease in their particle size range, causes a higher BET surface area of the geopolymer. The compressive strength of coal fly ash-based geopolymers can be controlled by waste glass addition.

The application of 20–30% of unwashed and unsorted waste glass (550.1 µm mean particle size) µm to the geopolymer matrix resulted in 65–60% higher specific surface area, 6–9% higher density, and 80% higher compressive strength compared to the unmodified samples. Furthermore, it is an economical and environmentally friendly solution to use this type of waste glass, due to the reduced energy consumption and costs associated with grinding or sorting raw materials.

Moreover, the results of leaching tests confirm that these types of materials can be classified as non-hazardous waste in accordance with the European Council Decision 2003/33/EC. Potential applications of the geopolymer composites described in this paper include materials for the manufacture of porous prefabricated elements, as well as materials for hardening outdoor pavings. Future research will focus on determining environmental and fire resistance for these types of materials.

## Figures and Tables

**Table 1:** Chemical composition of coal fly ash and waste glass.

Compound (%)	Material	
	Coal Fly Ash	Waste Glass
SiO_2_	48.22	73.40
Al_2_O_3_	26.13	1.43
Fe_2_O_3_	7.01	-
FeO	-	0.45
CaO	5.12	11.30
K_2_O	3.48	0.20
Na_2_O	1.62	11.96
MgO	1.72	1.25
SO_3_	1.11	-
TiO_2_	1.11	-
P_2_O_5_	0.70	-
MnO	0.090	-
Cl	0.09	-

**Table 2:** The composition of the evaluated geopolymer samples.

Designation of Samples	Composition			
	Waste Glass Quantities (%)	Waste Glass Particle Size (µm)	Coal Fly Ash (%)	Sand (%)
REF	-	-	90	10
A10	10	0.1–1200	80	10
B10	10	200–1200	80	10
C10	10	100–250	80	10
D10	10	63–120	80	10
E10	10	40–63	80	10
F10	10	0.1–40	80	10
A20	20	0.1–1200	70	10
B20	20	200–1200	70	10
C20	20	100–250	70	10
D20	20	63–120	70	10
E20	20	40–63	70	10
F20	20	0.1–40	70	10
A30	30	0.1–1200	60	10
B30	30	200–1200	60	10
C30	30	100–250	60	10
D30	30	63–120	60	10
E30	30	40–63	60	10
F30	30	0.1–40	60	10

## Data Availability

Not applicable.

## Supplementary Materials

The following supporting information can be downloaded at: https://www.mdpi.com/article/10.3390/ma16052044/s1.
