# Supplementary material for: Influence of Waste Glass Particle Size on the Physico-Mechanical Properties and Porosity of Foamed Geopolymer Composites Based on Coal Fly Ash
Source: Materials (Basel). 2023 Mar 1;16(5):2044. doi: 10.3390/ma16052044 (PMC10004531; doi:10.3390/ma16052044)
Supplement: Supplementary file 1 [file materials-16-02044-s001.zip › materials-2223427-supplementary.pdf]

Article

Influence of Waste Glass Particle Size on the Physico-Mechanical Properties and Porosity of Foamed Geopolymer Composites Based on Coal Fly Ash

Celina Ziejewska[1], Agnieszka Grela[2], Marek Hebda[1*]

[1] Faculty of Materials Engineering and Physics, Cracow University of Technology, Warszawska 24, 31-155 Cracow, Poland

[2] Faculty of Environmental and Power Engineering, Cracow University of Technology, Warszawska 24, 31-155 Cracow, Poland

* Correspondence: marek.hebda@pk.edu.pl (M.H.)

Supplementary Materials

Table S1. Particle size distributions of fly ash and waste glass powders.

Raw materials	D_{10} [µm]	D_{50} [µm]	D_{90} [µm]	Mean [µm]	Span $(D_{90}-D_{10})/D_{50}$ [µm]
Fly ash	2.3 ± 0.1	12.3 ± 1.3	37.0 ± 6.0	17.3 ± 2.5	2.8 ± 0.2
0.1-1200 WG (all fractions)	112.8 ± 8.6	483.4 ± 19.7	896.7 ± 51.1	550.1 ± 18.9	1.6 ± 0.2
200-1200 WG	303.1 ± 1.6	584.9 ± 4.4	1123.3 ± 5.5	702.4 ± 1.6	1.4 ± 0.02
100-250 WG	30.4 ± 1.5	155.2 ± 0.5	248.6 ± 0.2	160.6 ± 0.6	1.4 ± 0.01
63-120 WG	6.3 ± 0.1	55.4 ± 1.2	118.5 ± 1.2	60.8 ± 0.9	2.0 ± 0.02
40-63 WG	4.9 ± 0.1	33.3 ± 0.1	63.1 ± 0.2	35.1 ± 0.1	1.7 ± 0.01
0.1-40 WG	3.9 ± 0.8	19.8 ± 0.3	39.2 ± 0.2	22.0 ± 0.3	1.8 ± 0.2

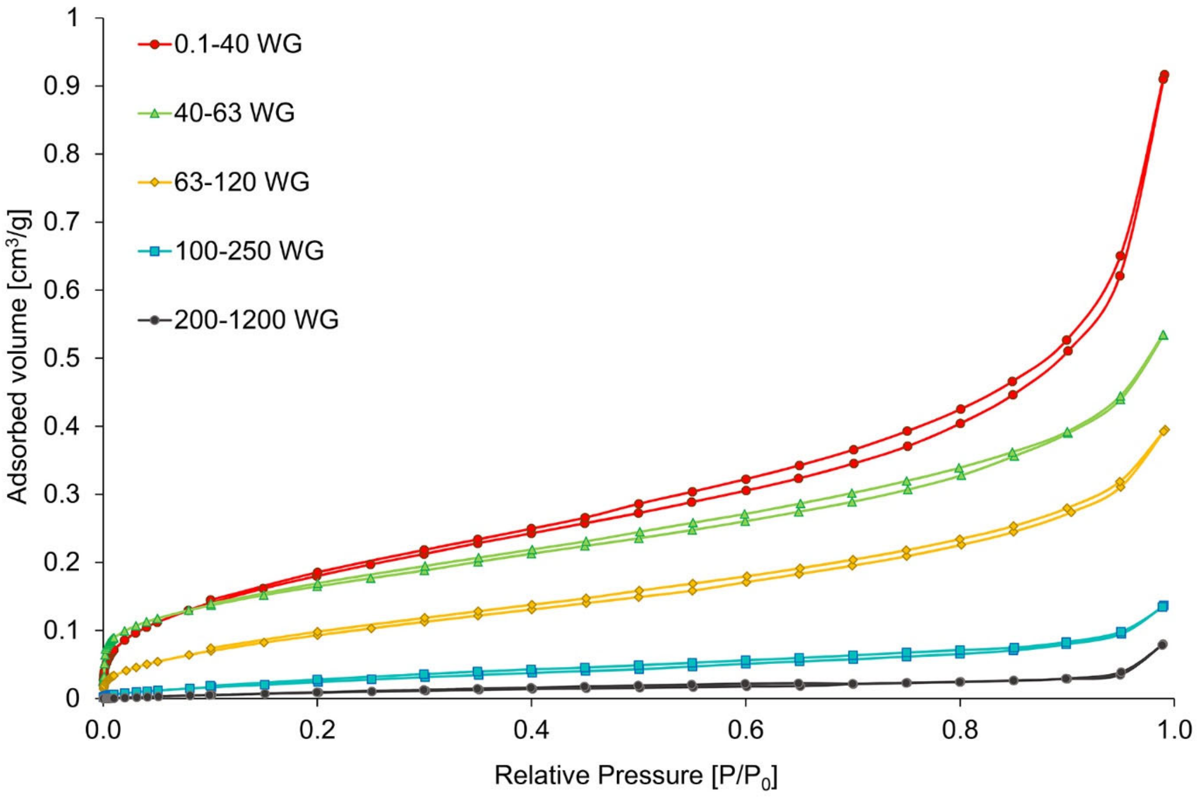

Figure S1. Nitrogen adsorption-desorption isotherms of waste glass after separation into five different fractions.

Table. S2. The specific surface area of geopolymers, depending on the used amount and particle diameters of waste glass.

Designation of samples	BET Surface area [m^2/g]
REF	22.772
A10	30.881
A20	37.556
A30	36.435
B30	16.089
D30	37.207
F30	43.711

Figure S2. The determination of porosity of geopolymers using ImageJ software. The best channel for analysed photography was chosen by means of the "Split Channels" option. After that, the maximum possible part of the image was selected using the rectangular section tool. The threshold tool was used to determine the porosity areas. Then the photograph was converted into a binary image. Finally, an analysis of particles option was chosen, and the obtained area fraction value was saved. All of the steps were repeated for each sample. The porosity results were the average of the image analysis of a minimum of three samples.

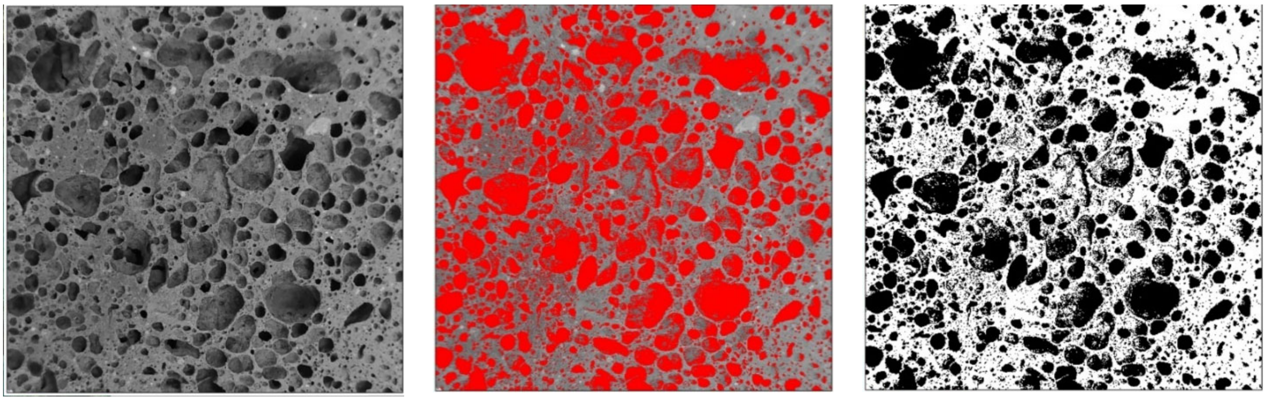

Table S3. The results of the leaching test conducted for geopolymer samples.

**Designation of samples**	**Unit**	**REF**	**A30**	**B30**	**D30**	**F30**
pH of the water extract	-	10.97 ± 0.08	10.84 ± 0.08	10.84 ± 0.08	10.83 ± 0.08	11.00 ± 0.08
total dissolved substances	mg/dm^3	5884 ± 231	5805 ± 228	5352 ± 210	6521 ± 256	8518 ± 334
chlorides		26.4 ± 2.2	11.2 ± 0.9	11.6 ± 1.0	11.9 ± 1.0	13.6 ± 1.1
fluorides		3.4 ± 0.3	2.7 ± 0.3	2.8 ± 0.3	2.3 ± 0.2	2.2 ± 0.2
sulphates		479 ± 39	335 ± 27	357 ± 29	349 ± 28	345 ± 28
Zn		0.0010 ± 0.0002	< 0.001	< 0.001	0.0050 ± 0.0008	0.0020 ± 0.0003
Cd		< 0.001	< 0.001	< 0.001	< 0.001	< 0.001
Cu		< 0.001	< 0.001	< 0.001	< 0.001	< 0.001
Pb		0.023 ± 0.003	0.040 ± 0.006	0.069 ± 0.010	0.066 ± 0.010	0.276 ± 0.040
Ni		0.0020 ± 0.0002	0.0030 ± 0.0004	0.0020 ± 0.0002	0.0030 ± 0.0004	0.0040 ± 0.0005
Ba		0.016 ± 0.004	0.006 ± 0.001	0.0020 ± 0.0004	0.0010 ± 0.0002	0.012 ± 0.0003
Cr		0.019 ± 0.004	0.010 ± 0.002	0.012 ± 0.003	0.012 ± 0.003	0.007 ± 0.002
Cr(VI)		< 0.020	< 0.020	< 0.020	< 0.020	< 0.020
Hg		< 0.01	< 0.01	< 0.01	0.06 ± 0.01	< 0.01
As		1.06 ± 0.11	0.72 ± 0.07	0.66 ± 0.07	0.80 ± 0.08	0.87 ± 0.09
Se		0.12 ± 0.02	0.09 ± 0.01	0.09 ± 0.01	0.10 ± 0.01	0.12 ± 0.02
Mo		0.43 ± 0.05	0.28 ± 0.03	0.26 ± 0.03	0.29 ± 0.03	0.30 ± 0.03
Sb		< 0.02	< 0.02	< 0.02	< 0.02	< 0.02
dissolved organic carbon		8.28 ± 0.75	7.75 ± 0.70	5.24 ± 0.48	7.53 ± 0.68	6.55 ± 0.60
